# Autonomous decisions by couples in reproductive care

**DOI:** 10.1186/s12910-020-00470-w

**Published:** 2020-04-25

**Authors:** Amal Matar, Anna T. Höglund, Pär Segerdahl, Ulrik Kihlbom

**Affiliations:** Centre for Research Ethics and Bioethics, Department of Public Health and Caring Sciences, Uppsala University, Box 564, 751 22 Uppsala, Sweden

**Keywords:** Couple autonomy, Expanded carrier screening, Preconception, Relational autonomy, Reproductive autonomy

## Abstract

**Background:**

Preconception Expanded Carrier Screening (ECS) is a genetic test offered to a general population or to couples who have no known risk of recessive and X-linked genetic diseases and are interested in becoming parents. A test may screen for carrier status of several autosomal recessive diseases at one go. Such a program has been piloted in the Netherlands and may become a reality in more European countries in the future. The ethical rationale for such tests is that they enhance reproductive autonomy. The dominant conception of autonomy is individual-based. However, at the clinic, people deciding on preconception ECS will be counselled *together* and are expected to make a joint decision, as *a couple*. The aim of the present study was to develop an understanding of autonomous decisions made by couples in the context of reproductive technologies in general and of preconception ECS in particular. Further, to shed light on what occurs in reproductive clinics and suggest concrete implications for healthcare professionals.

**Main text:**

Based on the shift in emphasis from individual autonomy to relational autonomy, a notion of *couple autonomy* was suggested and some features of this concept were outlined. First, that both partners are individually autonomous and that the decision is reached through a communicative process. In this process each partner should feel free to express his or her concerns and preferences, so no one partner dominates the discussion. Further, there should be adequate time for the couple to negotiate possible differences and conclude that the decision is right for them. The final decision should be reached through consensus of both partners without coercion, manipulation or miscommunication. Through concrete examples, the suggested notion of couple autonomy was applied to diverse clinical situations.

**Conclusions:**

A notion of couple autonomy can be fruitful for healthcare professionals by structuring their attention to and support of a couple who is required to make an autonomous joint decision concerning preconception ECS. A normative implication for healthcare staff is to allow the necessary time for decision-making and to promote a dialogue that can increase the power of the weaker part in a relationship.

## Background

*Partner A and partner B have been thinking of having a baby for a while, but they are worried. Partner A’s best friend Linda has recently given birth to a baby with X, a severe genetic disease. This was completely unexpected, as neither Linda nor her husband had a positive family history of the disease. Partner A and partner B plan a visit to their family doctor to discuss their concerns of begetting a child with a similar condition or another severe disease. Neither knows of a history of a genetic disease in their families. During their visit, the family doctor informs them about a preconception test for severe autosomal recessive genetic diseases and explains the test procedure, risks and benefits and the available reproductive options in case of positive results.*

The test offered in the above fictitious scenario is a preconception Expanded Carrier Screening (ECS) panel, which is a new genetic test approach offered to a general population without a known prior risk of autosomal and X-linked recessive genetic diseases. It can also be offered to couples who have no known risk of recessive genetic diseases in their families or communities and are interested in becoming parents. A test may screen for the carrier status of several hundred autosomal recessive (AR) diseases at one go [1]. The result of preconception ECS testing can be provided as a couple based result (e.g., as it is done in the Netherlands) or as individual results (e.g., in commercial tests). Such a program has been piloted in the Netherlands [2] and may become a reality in more European countries in the future.

Previously, preconception genetic testing was primarily offered to couples with positive family history of a recessive genetic disease, or to specific communities or regions with high prevalence of severe recessive genetic conditions, e.g., for Tay Sachs screening in Ashkenazi Jews and Thalassemia carrier screening in Cyprus and Sardinia [3]. Recently, however, the technology has become more reliable and affordable allowing governments to consider mass screening for carrier status among the population. Each of us carries on average 2.8 pathogenic mutations in autosomal recessive genes for severe diseases. These diseases are individually rare but taken together they result in significant portions of infant mortality (around 20%) and hospitalizations (around 18%) [1]. As carriers of a single recessive trait we are unaffected phenotypically. If a couple, such as partner A and partner B, undergoes preconception ECS and gets the result that they both carry the recessive gene for the disease X, then they are at 25% risk of having a child with the disease with each pregnancy [4].

Concerning the ethics of preconception ECS, the main rationale put forward in the bioethical literature for implementing preconception screening programs is that they would enhance reproductive autonomy [5]. The dominant conception of reproductive autonomy is individual-based, in the sense that autonomy is attributed to individuals and thus decisions made by individuals can be judged as autonomous or not [5, 6]. However, at the clinic, partner A and partner B and other couples taking a genetic test within a preconception ECS program will be counselled *together* and are expected to make a joint decision, as *a couple*. The literature is surprisingly silent on how couples can be expected to make a joint autonomous decision on reproductive related issues. Most contributions to the bioethical discussion on couples making reproductive decisions focus on parents as a single unit, without elaborating much on the fact that parents constitute two individuals who are in an interdependent committed relationship. Hence, one commonly finds words such as ‘parental responsibility’, ‘parental autonomy’, or ‘parental obligation’ without much discussion on the extent of the effect of the interrelationship and interdependency of these two individuals on their reproductive decision-making [7–9].

Nevertheless, there is much literature addressing the complexity of reproductive decision-making among partners in other disciplines, for instance, Decruyenaere et al. [10], Dommering et al. [11] and Hershberger and Pierce [12] identified factors contributing to parental reproductive decision-making when using new reproductive technologies such as parents’ perception of risk or past experiences of a genetic disease. Shehab et al. [13] examined the interaction between partners when they undertake a reproduction related decision. The decision was reached either intuitively and in consensus or following discussion and negotiations when a couple had opposing views.

These papers, however, neither discuss reproductive decisions in relation to reproductive autonomy, nor, in most cases, address the extent of each partner’s influence on autonomous reproductive decisions. One exception is Osamor and Grady [14] who discuss autonomy in relation to couples’ joint decision-making in healthcare. Their paper discusses heterosexual couples’ decisions in healthcare, with focus on the role and autonomy of the woman. They argue that the dynamics of couples’ joint decision-making exist on an autonomy spectrum. As women traditionally have less power in traditional marriages, the ethical value of a couple’s joint decision should be assessed against specific cultural, ethnic and religious backgrounds, according to them.

In our paper, we aim to develop an understanding of autonomous decisions made by couples in the context of reproductive technologies in general and of preconception ECS in particular. Preconception ECS is just one example that presents the issue of process that can result in a reproductive decision from a couple. In this paper, we propose the notion of *couple autonomy* and outline some features of this concept. We further suggest that the couple’s joint decision might be autonomous *at the level of the couple*.

Moreover, we attempt to shed light on what occurs in reproductive clinics and to suggest some concrete implications for the approach of healthcare professionals in those clinics. We believe the notion is pertinent to all situations where a couple is expected to express their reproductive autonomy, make a reproductive decision together and an informed consent is required from both of them.

## Main text

### Preconception ECS and reproductive autonomy

The Health Council of the Netherlands has defined reproductive autonomy as “the ability and opportunity to make one’s own, well-considered decisions concerning procreation” [15]. This is in line with how autonomy in medical ethics traditionally has been understood – as something that is expressed through well-informed and free choice of the individual. The choice through which you express your autonomy should be your “own”. To have your autonomy respected has, in corollary, been conceived as having the right to make well-informed decisions that reflect your own will [16, 17].

Reproductive autonomy as a *negative right* means that an individual has a right to non-interference from anyone (or the state) regarding his/her reproductive choices. Reproductive autonomy as a *positive right* entails that others (or the state) should offer procedures that enable an individual to make reproductive choices [18, 19]. When governments offer assisted reproductive technologies such as preconception ECS or in vitro fertilization (IVF), they are actively facilitating reproduction as a positive right. For example, in Sweden, up to three cycles of IVF financed by the healthcare system are offered to involuntary childless couples [20]. Other Nordic countries also offer fully funded assisted reproductive technologies [21, 22].

Reproductive autonomy has been associated with the offering of preconception ECS. According to de Wert and colleagues [5], preconception genetic screening programs should enhance reproductive autonomy by providing parents with more reproductive options and at the same time avoid some of the ethical dilemmas related to other techniques, e.g., abortion after prenatal screening. The reproductive options couples may encounter after a positive test result are several, e.g., they can have assisted fertilization by egg and/or sperm donation, preimplantation genetic diagnosis (PGD) and IVF, decide not to have a child, or simply accept the risk [5]. These concepts were reiterated in a study interviewing healthcare professionals in Sweden. Participants maintained that preconception ECS could enhance reproductive autonomy and reduce incidence of abortion [23]. Still, none of these articles elaborated on the role of the couple or the possibility of couple autonomy in reproductive decisions.

### Reproductive decisions made by couples

As with personal autonomy in general, the literature attributes reproductive autonomy to individuals rather than to couples or groups of people [24]. However, decisions whether to undergo preconception ECS or not are made by couples in the clinic, where they will also receive the results of the test and be counselled together so, how should this be understood?

One way to understand “a couple” is that at a particular moment in time two individuals are committing to make a reproductive decision together. They have decided to identify themselves as a couple, regardless of their legal status, for example, being married or cohabitating. Such individuals may have decided to reposition from other forms of relationships (friends, co-workers etc.) to a couple category, which, in our view, is distinctive because it can potentially result in reproductive related consequences.

The reproductive decision the couple undertakes together may result in the birth of a child who looks upon them as parents. And as such, there is a temporal dimension to the decision with potential for long-term effects. Whether the long-term effects are in the form of parental responsibility to care and provide for a healthy child or extra care for a diseased one, there is a commitment to shared responsibility. Moreover, individuals are forging new identities with such a decision, for example, being a couple or being a parent. Indeed, the offering of tests such as preconception ECS allows the couple time for reflection to plan a pregnancy and, arguably, better express their reproductive autonomy.

However, what has been perceived as the traditional conception of autonomy does not capture the nature of joint decisions made by parties imbedded in such relationships. Moreover, it has been under criticism for being too individualistic and not reflecting the way in which persons are imbedded in intimate relations with others. In theories on *relational autonomy* a common conviction is that “persons are socially embedded and that agents’ identities are formed within the context of social relationships and shaped by a complex of intersecting social determinants, such as race, class, gender, and ethnicity” [25, page 4].

### Weak and strong relational autonomy

In response to the criticism levelled against their conception of personal autonomy for being too individualistic, Beauchamp and Childress developed a view on autonomy that Anne Donchin has labelled *weak relational autonomy* [25, 26]. What makes Beauchamp and Childress’ view on autonomy relational, is that they concede that social aspects and personal relations are crucial conditions for self-development [17]. To illustrate this notion with the fictitious case of partner A and B, Beauchamp and Childress would probably acknowledge that the intimate relation, such as the one we suppose partner A and partner B have to each other, as well as relations to other persons, are crucial for their development of self-conception and agency. Moreover, Beauchamp and Childress would recognize that values, perceptions and aims can be shared with others and still properly considered as the agent’s own. So partner A and B may share the goal of having a child together and that goal can simultaneously be considered as partner A’s own goal or partner B’s own goal.

The reason why Donchin labels this view of autonomy as merely weakly relational is that the intimate personal relations acknowledged as being vital for self-development and agency can be, in principle, untied by choice [25, 26]. It is possible for partner B, on this view, to detach herself from others and the values she shares with them on the basis of her own will. In this sense partner B is still considered to be self-determined or independent and only contingently dependent on others. In making up her mind of having the preconception ECS test, partner B can, in principle, still arrive at her own decision without consulting partner A, according to this version of weak relational autonomy.

It seems fair to say that this notion of autonomy does not really address the question of conjoint decision-making. To treat partner A and partner B as two separate and merely contingently interdependent individuals in the context of preconception ECS may seem not only impractical, but also implausible. As a couple, they are likely to have strong emotional bonds between themselves, and it is reasonable to assume that they share a past and plan a future together that entails having a child together as well as taking responsibility for that child. Therefore, they together form a strong relational association and their autonomous decisions are necessarily inter-reliant and weak relational autonomy in this sense does not seem to capture this.

On Donchin’s notion of *strong relational autonomy*, and in contrast to weak relational autonomy, personal relations as well as shared values and aims are necessary parts of the autonomy of the agent [25, 26]. Partner A, for instance, is necessarily dependent upon partner B in several ways. A’s values, goals and reasoning must, in this case, be understood as involving partner B’s values and reasoning as well, and not only subjectively from partner A’s own perspective. The same goes for partner B.

Continuing with the strong conception of relational autonomy, societal practices and institutions influence the choice the agent makes. This means that what reproductive choice a person selects regarding preconception ECS are shaped by shared values and more general norms and practices. The example from preconception ECS might indicate that the couple in question is a man and a woman, but we argue that the arguments can extend and are thereby relevant to couples constituted in many alternative forms.

Autonomy in the strong relational sense is said to be both reciprocal and collaborative [25, 26]. The former attribute implies that partner A’s and partner B’s self-conception and autonomous will necessarily depend on their relations with each other, as well as with other people. This interdependence is also conceived as dynamic, in that it changes over time. The collaborative feature of strong relational autonomy means that we are dependent upon the support and influence of others in order to be able to make an autonomous choice. Partner A cannot be expected to make a fully autonomous decision without the support and influence of others, and in this case especially of partner B, and vice versa.

### Couple autonomy – three proposed demands

As Donchin points out, autonomy in the strong relational sense is still something that should be attributed to individuals rather than to collectives, such as the couple consisting of partner A and B [25, 26]. She does not provide an explicit reason for this claim, but it can reasonably be expected that treating couples or greater collectives as the smallest single decision-making unit, threatens to make power inequalities and undue decision-making processes invisible. A partner’s decision may be influenced by manipulation, persuasion or coercion [14].

However, we argue that even if partners A and B are autonomous as individuals, and even if their decision is properly “theirs” individually, the following still holds: if the decision was reached through an interactive process that satisfies certain demands, *then they are autonomous also as a couple*, and the decision is properly *the couple’s autonomous decision*. In other words, autonomy can, if certain demands are satisfied, be transferred from the individual level to the couple level, and be identified at both levels. This can be compared to how we think in relation to language. Individual words can have meaning, and when they are used in a sentence, the sentence can have meaning, too. Hence, linguistic meanings can exist both at the level of individual words and at the sentence level. We argue that the same way of thinking could be applied to autonomy, in individuals and in couples. Moreover, and as noted above, the clinical reality is that couples are sometimes expected to make joint decisions that in some sense should be autonomous.

The demands on couple autonomy that we propose here are not exhaustive, but are meant as suggestions that warrant further discussion and elaboration. In our view, a reproductive choice made by a couple is autonomous at the couple level if and only if:

1. Both partners are individually autonomous.
2. The decision is reached through a communicative process characterized by for instance:

- Each feels free to express his or her concerns and preferences so no one partner dominates the discussion, either by coercion or manipulation as defined in Osamor and Grady [14].
- There is adequate time for the couple to negotiate possible differences and conclude that the decision is right for them.
- The final decision is reached through consensus of both partners, where no persuasion may be used, as defined in Osamor and Grady [14].

3. One partner can autonomously transfer aspects of the decision to the other partner (e.g., permit some of the features above to be less prominent).

The notion of *couple autonomy* is a normative conception of autonomy in several respects. It is normative not only in that others, such as healthcare professionals, are required to assume responsibility to recognize the social context and the power inequalities which may impact couple autonomy and endeavor to repair autonomy failure of couples in their care. It is normative also in that partners to be fully autonomous as a couple should take on a responsibility toward demands of the kind we sketched above of couple autonomy. It is plausibly a responsibility to be supportive of your partner, to be transparent with regard to your own aims and goals when collaborating with your partner, and a responsibility to respect the goals and values of your partner. If such demands are fulfilled, it is reasonable to state that the decision reached is autonomous at *the couple level*.

This makes couple autonomy distinct from the relational concept of autonomy, which discusses and considers autonomy primarily at the individual level, where social, cultural and other factors influence a person’s autonomy and does not relate to two people coming together and making a joint decision in a healthcare setting.

It is important to point out that patterns of relationships are diverse and some of them may fall into categories that seem non-ideal or imperfect. In fact, most couples develop their understanding and devise their own equilibrium, and the role of healthcare professionals should not damage this understanding nor should they impose their moral ideal on the couple. The role of the healthcare professionals is to facilitate and enhance the expression of couples’ reproductive autonomy.

### Couple autonomy in the clinic

So, ideally, we may suppose that partner A and B in our fictitious example, understand the procedure’s risks vs. benefits and ask their genetic counsellor to give them some time to weigh their options and decide if they wish to undergo preconception ECS or not. They take their time to discuss and deliberate together over the information given. They may have differing views regarding the reproductive options available, if they both happen to be carriers of the same severe genetic disease. For example, partner A is against sperm donation for IVF and partner B is uncomfortable with a decision opting for an abortion. But through a transparent and respectful dialogue, they finally reach a solution that is agreeable to both. They might decide to undergo preconception ECS and will not consider sperm or egg donation and abortion as options if the test proves they are both carrier of the same severe genetic disease, thereby respecting both opinions. This case seems to fulfil the demands we proposed for couple autonomy. They express their concerns and preferences, they give themselves the time they need to make the decision and finally reach consensus regarding their reproductive choice. This might not always be the case, which will be further elaborated in two fictitious examples below.

For a couple as has been defined earlier, i.e., sharing strong emotional bonds, future goals and history, the relational influence will be strongly affecting their autonomy. It is worth noting that everything considered, it can be argued that an individual has mostly control over who, when and how much effect their partner have on their decisions. This is important in order to distinguish relational autonomous decisions from coercive influence. However, we argue that there would always be some degree of relational effect. Zeiler [6] has referred to external and internal factors affecting one’s autonomous decisions.

In an ideal case, the autonomy of the couple can be said to be a joint responsibility to show respect towards themselves and the other. A breach of couple autonomy is when the individuals do not respect each other and one person’s reproductive autonomy is deemed more important than their partner’s. To illustrate this point, we will present two fictitious scenarios in which couple’s autonomous decisions are highlighted and discussed in light of the demands for couple autonomy presented above.

#### Example 1

*Partner C, a high caliber professional, has always made decisions regarding the family and rarely discusses the decisions with the partner before executing them. Partner D, who is not working and barely finished high school education, never opposes this. Sometimes, partner D nurtures different opinions, however, rarely expresses them because D minds the hassle and the long arguments, which do not necessarily end in best favor. In the clinic, partner D would rather test for severe diseases that might lead to sustained severe pain in the child. But partner C was asking all the questions and on a couple of occasions was dismissive of partner D’s concerns when D tried to express them.*

In the scenario of Example 1, there is an obvious inequality in power and education between the partners. One person is dominating and monopolizing the decision-making in the relationship and minimally values the partner’s input. The first condition for couple autonomy thus is not satisfied since arguably partner D is not allowed to be autonomous. Moreover, the interactive process is lacking an open discussion of concerns and preferences and the decision made is not agreeable to both partners.

Should the healthcare professional interfere in this situation or not? We think yes, the healthcare professionals can provide the platform for partner D to express reproductive autonomy. They can set an appointment with partner D and allow this partner the time and space to express concerns and preferences. In a way, the healthcare professional is redeeming partner D’s individual reproductive autonomy (first demand in couple autonomy) before facilitating a communication process between the partners that fulfils criteria a, b and c mentioned above by arranging another appointment between partner C and D.

#### Example 2

*Partner F‘s job requires that s/he is away for long periods of time, F wants to have a baby but the risk of having one with severe genetic disease, is putting partner F off pregnancy. A baby with genetic diseases is a big responsibility and partner F, by virtue of her/his job, cannot be there to support partner E. Preconception ECS appears to offer a solution. Partner E wants to be a parent and agrees with whatever partner F decides. There are no real objections or preferences for a particular choice on E’s part and E thinks F should make the decision about the test.*

Here in Example 2, we may see a conflict and power inequality interplay. But arguably partner E is embracing and accepting to F’s decision. There is an agreement by the couple of who makes the final decision regarding preconception ECS. Partner E is transferring most aspects of the decision-making and consequently the responsibility associated with the decision to the other partner and has no objections to the decision partner F undertakes. Partner E and F, it can be argued are individually autonomous. There was an open communication process, which resulted in a consensus regarding transferring aspects of the decision-making to one partner, without any manipulation or coercion. Should the healthcare professional interfere in such a situation and give space and time for partner E to express their reproductive autonomy? Would this be considered as asking the healthcare professional to interfere in the acceptable arrangements between couples? Is it part of their professional responsibility to question such arrangements and the assignment of responsibility between the couple?

We think not. There is no coercion, manipulation or obvious miscommunication in the matter and the couple are both accepting the terms associated with the decision-making, unlike in Example 1 where partner C is dismissive of partner D’s concerns and views and partner D is too timid to express them. Partner D, in Example 1, is giving in to domination by partner C with respect to deciding on preconception test.

### Healthcare professionals and couple autonomy

The examples show that healthcare professionals might face ethical challenges when striving to enable couples to make relational autonomous decisions. They may encounter situations where one partner finds it difficult to be autonomous and express their choices because they are situated in a relationship (Example 1). If it were a single parent, the healthcare professional would focus on the parent to express an autonomous decision by full disclosure of information regarding the test, procedures and options available for the parent after testing.

At a first glance, the power inequalities may seem similar in the proposed scenarios; there is a dominant decision maker (in Example 1 and Example 2). However, in Example 2, partner E is consenting and embracing the fact that partner F is taking charge of the situation. There seems to be mutual respect and acknowledgment for each other’s preferences. In contrast, in Example 1, partner C exhibits domineering attitude towards partner D and ignores concerns and wishes. In other words, partner C shows little respect and overrides partner D’s expression of choices and autonomy.

In line with the notion of couple autonomy that we suggested above, the couple is situated in a web of relationships and their reproductive choice can be seen as a cooperative venture because of how they are currently situated and embedded in this partnership. Consequently, an autonomous decision for a couple deciding whether or not to undergo preconception ECS, must not be framed within an understanding of autonomy as non-interference or a strive for doing good to “undifferentiated others” [26]. Rather, this context displays the integration of lived experiences the parts of the couple share with each other and into which the healthcare professional is temporarily included.

The normative implication of this view is that healthcare professionals should encourage allocation of adequate time as well as dialogue and negotiation within the couple. However, the challenge of a relational approach of couple’s autonomy is to account for power differences in various relationships, not least in relation to societal norms for, e.g., gender, ethnicity and sexuality. It has been argued that significant others may have divergent values and priorities; hence, the significant others’ involvement in medical decision-making could counter patient autonomy and the patient’s best interest [27]. Against this it has been argued, that a relational approach to autonomy can make conflicts of interest within a relationship visible and promote a dialogue and a negotiation that could strengthen the autonomy of all parts in a decision-making process [28].

We argue, that by recognizing how intimate relations to others necessarily enter into one’s self-conception and evaluative outlook, a notion for couple autonomy can, arguably, be fruitful for healthcare professionals by structuring their attention to and support of a couple who is required to make an autonomous joint decision. The promising part of seeing a couple in a framework of couple autonomy is the opportunity to allow the necessary time for decision-making and to promote a dialogue that can increase the power of the weaker part in the relationship.

In practice, clinicians may not be able to easily discern the differences in power interplay between Example 1 and Example 2. They may suggest a separate meeting with the seemingly less powerful partner and give them a chance to voice their concerns if any exist. The healthcare provider ideally should support the couples to express their individual autonomy if possible. Yet, when it comes to preconception choices and decisions it is often the case that it is a couple that is to decide, and our suggested version of couple autonomy can form a starting point for a clinical practice where also couples, not only individuals, are seen as autonomous.

Compared to the arguing of Osamor and Grady (14) our suggestion goes further, in that we have developed the concept of couple autonomy and proposed three demands for it to be present. Thereby, we argue that we provide useful tools for the healthcare staff, who are to assess whether a joint decision by a couple is autonomous or not and ethically acceptable or not. Couple autonomy is thereby not only a philosophical concept, but also a practical notion that can be useful for healthcare professionals in the clinic, when they are to support decisions that are required to be made jointly, and not individually, by a couple.

## Conclusions

In context of some reproductive decisions, it is not one partner that is making the decision but rather a couple, and thereby the individual conception of autonomy is not applicable. The couple shares in this context a responsibility in making a conjoint decision and a responsibility towards each other to be transparent regarding their individual goals and values. As a couple reaching a joint, autonomous decision, they should respect each other in terms of will, goals, and values during the decision-making process. Moreover, healthcare professionals have new types of responsibility. They should facilitate the process to ensure the expression of the couple’s reproductive autonomy and assist them in reaching an autonomous decision at the couple level.

This article has proposed a notion of couple autonomy. If certain demands are met, couple’s reproductive decision can be accepted by healthcare staff as autonomous. The suggested demands on couple autonomy include that both partners are individually autonomous and that the decision is reached through a communication process which enables expression of concerns and preferences by each partner and free of coercion, manipulation and miscommunication. Further, the decision-making process should allow them enough time to weigh options and reach a decision that feels right for both parties; and, lastly, there is consensus over the final decision. In certain cases, one partner can autonomously transfer some aspects of the decision to the other partner. This characterization of couple autonomy aims to help resolve some of real life scenarios occurring inside reproductive clinics, where the individualistic conception of personal autonomy may seem both impractical and implausible.

## Data Availability

Not applicable.

## Abbreviations

ECS: Expanded Carrier Screening
AR: Autosomal Recessive
IVF: In vitro fertilization
PGD: Preimplantation Genetic Diagnosis
